# Myxedema

**Published:** 1888-11

**Authors:** 


BUFFALO MEDICAL AND SURGICAL JOURNAL
A MONTHLY REVIEW OF MEDICINE AND SURGERY.

EDITORS:
THOS. LOTHROP, M. D., - - - W. W. POTTER, M. D.

All communications, whether of a literary or business character, should be addressed to the editors: 284 FRANKLIN STREET, BUFFALO, N. Y.

### Editorial.

### MYXEDEMA.

Two important studies of this peculiar disease have recently been made—one by a committee appointed by the Clinical Society of London, and the other by Dr. Henry Hun, of Albany. The one study was quite independent of the other, and both taken together form the most valuable contribution ever made to the subject. The committee of the Clinical Society of London had been prosecuting experiments and researches for a period of nearly four years, and its conclusions were presented to the Society last May through its chairman, Dr. W. M. Ord. Dr. Hun gave the results of his investigations in a paper published in the *American Journal of the Medical Sciences* for July and August, 1888, reporting therein four cases of his own, and analyzing 150 cases reported by others.

Grouping the conclusions of these statistics together, it appears that myxedema is practically the same disease as that named "sporadic cretinism;" is "probably identical with cachexia strumipriva;" and bears a close affinity to "endemic cretinism."

Clinical and pathological observations indicate that *destructive change, either by removal or disease of the thyroid gland, is the one condition common to all cases.* The most common form of destructive change of the thyroid consists "in the substitution of a delicate fibrous tissue for the proper glandular structure,"—a nearly complete atrophy of the parenchyma of the gland, and in some cases, at least, a new formation of lymphatic tissue in it. Other lesions, which are more or less common, are a general obliterating endarteritis, with consequent hypertrophy of the left side of the heart, a chronic diffuse nephritis, an interstitial hepatitis, degeneration of the suprarenal capsules, atrophy of the fat, and a general edema or infiltration of the skin and mucous membranes.

Patients of all ages may be affected with the disease, but it is most frequently found between the ages of thirty-five and forty years. It is more common in females than males, and excessive child-bearing, exhaustive hemorrhages, mental shock, worry, and injuries are important etiological factors. It may develop insidiously, or commence with some nervous or cutaneous disease.

The symptoms of myxedema are very characteristic, and affect especially the skin and mucous membranes, and the nervous and vascular systems. Dr. Hun summarizes them very clearly as follows: "The skin is swollen without pitting, dry, scaly, and cold, the hair and teeth frequently fall out, the nails become brittle, and the perspiration is either greatly diminished or absent. The mucous membranes are also swollen, but their secretion is usually increased. There is mental sluggishness and impairment, and insanity is frequent; sensibility, both special and general, is impaired in about half the cases; the muscles act feebly and sluggishly in all cases; the reflex actions are frequently diminished; speech is slow, and in more than half the cases hoarse; and numbness and neuralgic pains are frequently present. In the majority of cases the pulse is slow and small, and the heart presents some abnormality. The blood is often in an anemic condition, and very frequently there are severe hemorrhages. The temperature, especially the surface temperature, is sub-normal, which may be considered in part a nervous symptom."

The investigations of both the committee and Dr. Hun agree that mucin in the tissues is not in excess, as was believed by those who first made chemical observations on the disease.

Myxedema usually lasts a number of years—sometimes as many as fifteen or twenty. Very few cases fully recover, and most of them die sooner or later, usually from some intercurrent or associated affection. The course of the disease is subject to considerable variations, the swelling of the skin and other symptoms being much worse at certain times than at others.

Regarding treatment, the authors have very little to say. Among the remedies which Dr. Hun mentions are nitro-glycerine, digitalis, iron, strychnia, jaborandi, electricity, baths and friction.

Myxedema is of great physiological, as well as of pathological, interest, for it shows, although in a far too indefinite manner, the very great influence which the thyroid gland has over the animal economy; and the enlightenment which the authors have given to the profession in thus presenting the results of their experiments, examinations, and researches will, in a measure at least, assist in penetrating the darkness which surrounds the very important function of this gland. We commend these studies to the profession as the most complete and trustworthy of any that have yet appeared, on the symptoms and pathology of this most interesting and peculiar disease.

